# Vendor density mapping and compliance assessment with tobacco control laws around schools in Bhubaneswar City—a geo-spatial mapping and observational study

**DOI:** 10.3389/fpubh.2025.1410114

**Published:** 2025-06-13

**Authors:** Nancy Satpathy, Pratap Jena, Amit Yadav, Venkatarao Epari, Vikrant Mohanty, Muhammad Imran Ali, Rishika Khare, Yogesh Pratap Singh, Ashish Kumar Pandey

**Affiliations:** ^1^Department of Community Medicine, Institute of Medical Sciences and SUM Hospital, Siksha O Anusandhan University, Bhubaneswar, India; ^2^Indian Council of Medical Research (ICMR), New Delhi, India; ^3^School of Public Health, Kalinga Institute of Industrial Technology (KIIT), Bhubaneswar, India; ^4^Swiss School of Business and Management, Geneva, Switzerland; ^5^Vital Strategies, Inc., New Delhi, India; ^6^Maulana Azad Institute of Dental Sciences, University of Delhi, New Delhi, India; ^7^Salaam Jeevan, Bhubaneswar, India; ^8^National Law University, Odisha, India; ^9^National Law University, Tripura, India; ^10^Vital Strategies, New York, NY, United States

**Keywords:** tobacco control, geo-spatial mapping, vendor density, COTPA Act, compliance

## Abstract

**Background:**

Tobacco use among youth remains a significant public health challenge, particularly in India, where vendor accessibility plays a crucial role in initiation and consumption. This study examines tobacco vendor density around schools in Bhubaneswar City, Odisha, utilizing advanced geo-spatial mapping techniques to provide evidence for regulatory enforcement.

**Methods:**

A geo-spatial mapping approach was employed using ArcMap 10.8 and Google Maps to identify tobacco vendors within a 100-yard radius of 15 selected high schools. Data collection was conducted through a structured questionnaire with 53 closed-ended questions via the Epicollect5 platform. The study adopted a probability proportional-to-size sampling method to ensure representative vendor distribution.

**Results:**

The study identified 107 tobacco vendors surrounding the selected schools, with an average vendor density of approximately seven per school vicinity. Pan vendors and grocery/convenience stores were the most prevalent vendor types. Despite existing regulations, widespread tobacco advertising, brand displays, and promotional activities were observed. Additionally, violations related to smoking near schools and sales to minors indicated gaps in regulatory compliance.

**Conclusion:**

The high density of tobacco vendors near schools underscores the need for strengthened enforcement mechanisms and policy interventions. Enhancing regulatory compliance through stricter zoning laws, targeted monitoring, and community-driven initiatives is essential to reducing youth exposure to tobacco products and mitigating associated health risks.

## 1 Introduction

Tobacco use among youth and children remains a pressing global public health crisis with far-reaching consequences (1). According to global estimates, one in every 10 girls and one in every five boys aged 13–15 years use tobacco (2). While tobacco consumption affects individuals of all ages, initiating use during childhood and adolescence is particularly detrimental, increasing the risk of long-term addiction, chronic diseases, and premature mortality (3). Its widespread use is a leading contributor to preventable death and disease, spanning a spectrum of chronic conditions such as cancer, respiratory ailments, cardiovascular disorders, and stroke (3).

Recognizing its detrimental effects, the World Health Organization (WHO) identifies tobacco use as a major risk factor for non-communicable diseases (NCDs), responsible for an estimated 8 million deaths annually (4, 5). This epidemic affects individuals of all ages and socioeconomic backgrounds, with a disproportionate impact on low- and middle-income countries (LMICs), where tobacco control measures may be less robust and more aggressive industry tactics (4, 6).

India, one of the world’s largest consumers of tobacco, faces an immense burden of tobacco-related morbidity and mortality. With nearly 267 million adult tobacco users, the country accounts for a significant share of global tobacco consumption (7). The prevalence of both smoking and smokeless tobacco products—such as cigarettes, bidis, hookah, khaini, gutkha, betel quid with tobacco, and zarda—makes tobacco control particularly complex (8). Of particular concern is the early initiation of tobacco use among Indian youth. Data from the Global Youth Tobacco Survey (GYTS) 2019 reveals that 38% of cigarette smokers, 47% of bidi smokers, and 52% of smokeless tobacco users in India began using tobacco before the age of 10. The median age at initiation for cigarette and bidi smoking was 11.5 years and 10.5 years, respectively, highlighting early onset of tobacco use. Additionally, nearly one-fifth of students aged 13–15 reported using some form of tobacco product in their lifetime, with a current usage rate of 8.5% in the last 30 days (9). These statistics emphasize the need for effective tobacco control measures to address early initiation and prevent tobacco use among youth. Early initiation of tobacco use is a risk factor for long-term addiction and adverse health consequences, emphasizing the need for targeted interventions to protect youth and children (10). Tobacco vendor density plays a crucial role in shaping tobacco consumption patterns, particularly among youth. Studies indicate that a high concentration of tobacco vendors near schools increases exposure and accessibility to tobacco products, significantly influencing initiation and continued use among students (11). Vendor clustering in school zones normalizes tobacco use, making it more socially acceptable and easier for minors to obtain tobacco products despite regulatory restrictions. Evidence from international and national research suggests that reducing vendor density near educational institutions can effectively lower youth smoking rates and prevent early initiation (11, 12).

Despite existing tobacco control laws, tobacco vendor density remains a largely under-researched aspect of youth tobacco prevention in India. Many studies focus on individual behavior, school-based interventions, or advertising restrictions, but fewer address how vendor proximity influences youth access and experimentation with tobacco (13). This study seeks to address this gap by systematically examining the density of tobacco vendors around schools in Bhubaneswar, Odisha. Understanding the geographical clustering of vendors can help policymakers strengthen zoning laws, restrict tobacco sales near schools, and implement targeted enforcement strategies.

Odisha, situated in eastern India, faces unique challenges regarding tobacco control. Data from the Global Youth Tobacco Survey (GYTS) 2019 reveals concerning prevalence rates of tobacco use among students in Odisha, with smokeless tobacco being the predominant form of consumption (14). Additionally, accessibility to tobacco products through tobacco vendors and exposure to tobacco advertising at points of sale present significant obstacles to effective tobacco control efforts in the state. Tobacco vendor density in Odisha remains high, with limited studies exploring its direct impact on youth tobacco use, making this an important area for research.

The Cigarettes and Other Tobacco Products Act (COTPA), enacted in 2003, serves as India’s primary tobacco control legislation, imposing restrictions on tobacco sales, advertising, and consumption in public places (15). COTPA includes key provisions such as Section 4 (prohibiting smoking in public places), Section 5 (banning tobacco advertising and promotion), and Section 6 (restricting tobacco sales to and by minors). However, compliance with these regulations remains suboptimal, particularly around educational institutions where students are highly vulnerable to tobacco exposure (16, 17). Weak enforcement of COTPA provisions allows the continued operation of tobacco vendors near schools, counteracting efforts to protect youth from early tobacco initiation.

The implementation of Tobacco-Free Educational Institution (TOFEI) guidelines has been pivotal in reducing tobacco use among students. A study in Maharashtra demonstrated that schools with trained teachers showed higher compliance with TOFEI criteria, leading to a significant decrease in tobacco consumption among students (18). Similarly, research in Puducherry revealed that schools adhering to TOFEI guidelines had reduced evidence of tobacco use on premises, highlighting the guidelines’ effectiveness in promoting a tobacco-free environment (19). These findings emphasize the importance of strict enforcement and regular monitoring of TOFEI guidelines to safeguard youth from tobacco exposure. Despite these efforts, vendor density around schools continues to undermine the effectiveness of COTPA and TOFEI policies, necessitating a comprehensive strategy that integrates vendor regulation with school-based interventions.

The study aims to map tobacco vendor density around 100-yard (91.44 meters) radius of schools and assess compliance with tobacco control laws in Bhubaneswar City, Odisha, India. By fulfilling these objectives, the study endeavors to provide valuable insights for informing evidence-based to support stricter zoning regulations and targeted interventions, ultimately contributing to more effective tobacco control policies for protecting youth.

## 2 Materials and methods

### 2.1 Study design and sampling

**Figure 1:**
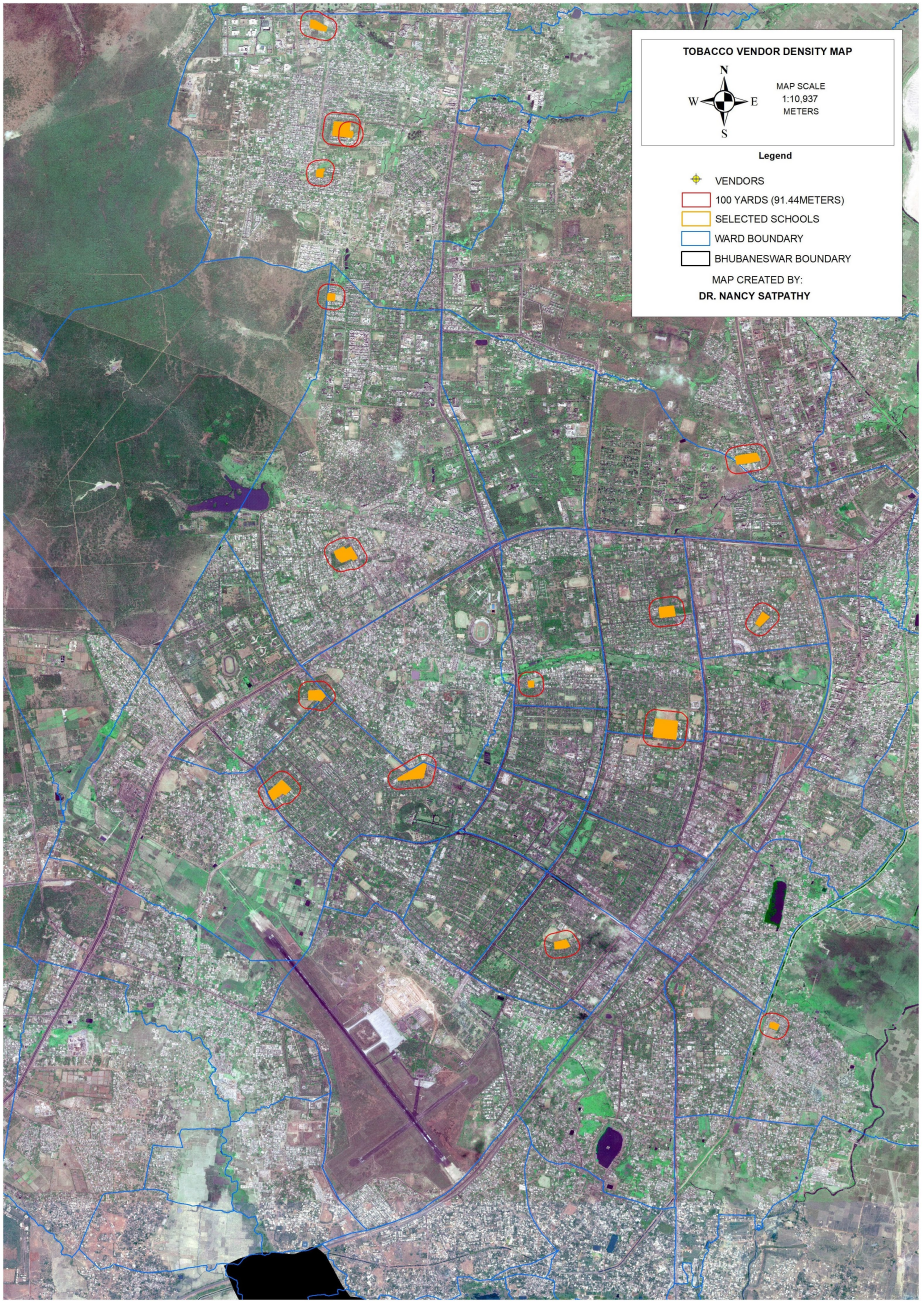
Mapping of all selected schools and a 100-yard zone around the schools.

This cross-sectional observational study, conducted as part of a doctoral research, aimed to evaluate tobacco vendor density and compliance with tobacco control laws within schools and their proximity areas in Bhubaneswar, Odisha, India, from November 2023 to January 2024.Geographically, Bhubaneswar is divided into three zones by the Bhubaneswar Municipal Corporation (BMC): north, southeast, and southwest (20). From a pool of 65 high schools listed under the Department of School and Mass Education, Government of Odisha (21), 15 high schools were selected as part of doctoral research. High school in India refers to classes 8–10 (Figure 1).

The selection was made using the probability-proportional-to-size (PPS) sampling method to ensure a representative sample aligned with the study’s precision and confidence level requirements, from a total of 65 high schools and a student population of 24,071. The sample size selection process was based on a formula utilizing a 95% confidence level, ±1.24% margin of error, and a prevalence of tobacco use in Odisha of 6.2% according to the Global Youth Tobacco Survey (GYTS), employing the P/5 approach for precision. Ethical clearance for the study was obtained from the institutional ethics committee of Siksha “O” Anusandhan, deemed to be University, Bhubaneswar, Odisha India, (Ref: Letter No.: Ref. No./DMR/IMS.SH/SOA/2021026).

### 2.2 Data collection and instrument

The identification of tobacco vendors within a 100-yard radius of the selected high schools was facilitated using advanced mapping software, namely ArcGIS version 10.8, complemented by Google Maps and satellite imagery to pinpoint significant landmarks and roads. Vendor selection criteria were formed by the types identified in the Global Youth Tobacco Survey (GYTS) (14), Global Adult Tobacco Survey (GATS) (22, 23) and insights from local stakeholders, encompassing small grocery stores, paan (betel leaf) and bidi (hand-rolled cigarette) vendors, street vendors, and tobacco specialists.

### 2.3 Questionnaire design and validation

A comprehensive self-designed, structured, and self-administered questionnaire comprising 53 closed-ended questions was developed in the English language to evaluate various tobacco-related activities in each outlet. The questionnaire was administered using the Epicollect5 platform, a free and easy-to-use mobile data-gathering platform and publicly available at https://five.epicollect.net. Key components from the COTPA Act, insights from Feighery et al. (22), and variables from the Global Youth Tobacco Survey (GYTS) and Global Adult Tobacco Survey (GATS) (23) were incorporated into the survey instrument. These variables encompassed aspects such as vendor types, advertisement types, branding practices, health warnings, compliance measures, and factors related to tobacco sales to minors, ensuring a comprehensive assessment of tobacco marketing practices and regulations (Supplementary File 1).

For reliability of the questionnaire, reliability analysis performed using SPSS, yielded a Cronbach Alpha coefficient of 0.797, demonstrating satisfactory internal consistency. The validity of the questionnaire was assessed through expert review to ensure clarity, understandability, and logical ordering of questions. Content validity was ensured by subjecting the questionnaire to scrutiny by experts involved in tobacco cessation activities, while face validity was assessed through feedback from these experts to ascertain the comprehensibility and relevance of the questionnaire content.

### 2.4 Data analysis

Descriptive frequency analysis was performed to analyze various vendor characteristics and compliance levels. Additionally, bivariate Chi-square analysis was conducted using IBM SPSS Statistics 25 to examine associations between government and private schools.

## 3 Results

### 3.1 Characteristics of tobacco vendors

**Figure 2:**
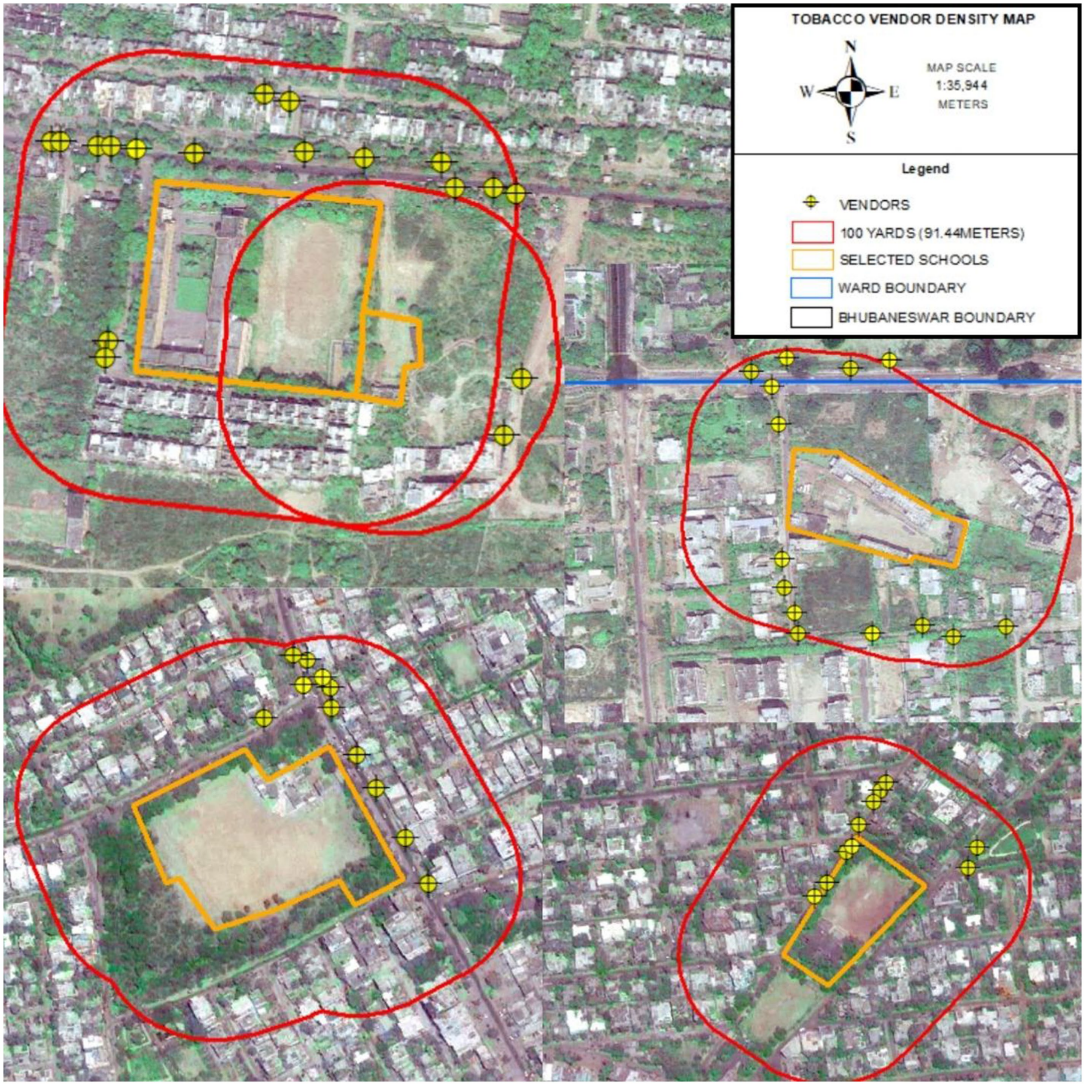
Geographic Information System (GIS) mapping of the 100-yard radius around five schools and the locations of tobacco vendors within this radius.

The study investigated various vendor characteristics and levels compliance among 226 vendors located within 100 yards of schools. Out of these 226 vendors, 107 were identified as selling tobacco products. The average density/presence of tobacco vendors within 100 yards of the school premises are approximately 07 (6.68) (Figure 2).

### 3.2 Types of vendors

Among these vendors, pan vendors represented the majority (44.9%), followed by grocery/convenience stores (36.4%), tea stalls (8.4%), large stores/supermarkets (6.5%), and mobile vendors (3.7%) (Table 1). Notably, all 15 selected schools had nearby tobacco-selling vendors, except for one government school, indicating the widespread nature of the lapse.

**Table 1:** Vendor Characteristics around 100 yards of schools (*n* = 107).

Variables	Govt schools (n)	(%)	Private schools (n)	(%)	Total (n)	(%)
Pan vendors	24	44.9	24	45.3	48	44.9
Tea stall	5	9.3	4	7.5	9	8.4
Grocery/convenience store	20	37	19	35.8	39	36.4
Large store/supermarket	4	7.4	3	5.7	7	6.5
Mobile vendor	1	1.9	3	5.7	4	3.7

### 3.3 Advertisement practices

In terms of advertisements in tobacco vendors, various types of advertisements were prevalent, including boards (15.9%), posters (22.4%), banners (8.4%), stickers (50.5%), dangles (33.6%), LCD/video screening/LED (12.1%), promotional gifts/offers (8.4%), and product displays (35.5%). Additionally, 48 (44.9%) vendors displayed brand pack shots or brand names of tobacco products, and 38 (34.6%) vendors used particular colors and layouts associated with specific tobacco products. Hoarding advertising of tobacco products larger than (60 cm × 45 cm) at the point of sale and more than two boards at the point of sale, was observed in 46 (43%) of vendors. Advertisement locations varied, with 26 (24.3%) placed in the exteriors and 50 (46.7%) placed inside the vendors. Advertisements were predominantly placed above 3 feet (28%) and below 3 feet (32.7%), while a smaller proportion was placed next to candy (12.1%).

Regarding health warning messages, compliance was suboptimal, with only 31 (29%) vendors displaying board/banner/poster health warnings as mandated by COTPA. Sixty one vendors, (57%) displayed tobacco brand names (Table 2).

**Table 2:** Compliance of tobacco vendors with COTPA section 5 of advertisement at point-of-sale around 100 yards of schools (*n* = 107).

Variables	Govt schools (n)	(%)	Private schools (n)	(%)	Total (n)	(%)
Advertisements in tobacco vendors	30	55.6	31	58.5	61	57
Type of advertisements—boards	10	18.5	7	13.2	17	15.9
Type of advertisements—posters	15	27.8	9	17	24	22.4
Type of advertisements—banners	6	11.1	3	5.7	9	8.4
Type of advertisements—stickers	26	48.1	29	54.7	55	50.5
Type of advertisements—dangles	20	37	16	30.2	36	33.6
Type of advertisements—LCD/video screening/LED	6	11.1	7	13.2	13	12.1
Type of advertisements—Promotional gifts/offers	6	11.1	3	5.7	9	8.4
Type of advertisements—product display	19	35.2	19	35.8	38	35.5
Advertisement board displays brand packshot or brand name of tobacco products	24	44.4	24	45.3	48	44.9
Whether the particular color and layout and/or presentation is used in an advertisement board that is associated to particular tobacco products	17	31.5	21	39.6	38	34.6
Presence of hoarding advertising tobacco products, larger than (60 cm × 45 cm) at point of sale and more than two boards at point of sale	24	44.4	22	41.5	46	43
Advertisement location-exterior	12	22.2	14	26.4	26	24.3
Advertisement location-interior	24	44.4	26	49.1	50	46.7
Advertisement placement- below 3 feet	19	35.2	16	30.2	35	32.7
Advertisement Placement- Next to Candy	8	14.8	5	9.4	13	12.1
Advertisement Placement-Above 3 feet	14	25.9	16	30.2	30	28
Presence of board/banner/poster displays a health warning	16	29.6	15	28.3	31	29
Whether health warning is on uppermost portion of a board	16	29.6	15	28.3	31	29
Whether health warning is written in any local Indian language (and/or English)	16	29.6	15	28.3	31	29
Name of the tobacco brand displayed?	28	51.9	29	54.7	61	57

### 3.4 Smoking in public places and sales to minors

Regarding smoking in public places, an alarming 62 (57.9%) of vendors allowed smoking within 100 yards of schools, posing a significant public health challenge, particularly given their proximity to educational institutions. Signage near schools needed to be improved, with only 6 (5.6%) vendors displaying signage as required by law. The study also uncovered concerning trends related to tobacco sales to minors, with 52 (48.6%) vendors selling tobacco products to minors and 26 (24.3%) having tobacco products sold by minors (Supplementary File 2). These findings underscore the need for stringent enforcement measures to prevent youth access to tobacco products and protect minors from the harms of tobacco use (Table 3).

**Table 3:** Compliance of COTPA section 4 and section 6 regulations (*n* = 107).

Variables	Govt schools (n)	(%)	Private schools (n)	(%)	Total (n)	(%)
Presence of smoking 100 yards of educational institution	35	64.8	27	50.9	62	57.9
Signage near educational institutions (100 yards)	10	18.5	11	20.8	21	19.6
Display of signage as mandated in law 6(a) of COTPA	3	5.6	3	5.7	6	5.6
Tobacco products are sold by minors	12	22.2	14	26.4	26	24.3
Tobacco products are sold to minors	25	46.3	27	50.9	52	48.6

### 3.5 Brand names displayed

The most commonly displayed tobacco brand names as direct adverstisements were Gold Flake (15%), Marlboro (23.4%), Classic (15.9%), Wills (3.7%), and Four Square (8.4%). Indirect advertisement practices also exhibited similar trends that included boards (15%), posters (36.4%), banners (29%), stickers (32.7%), and dangles (38.3%). Notably, tobacco brand names such as Vimal (3.7%), Bahar (29.9%), Pan Bahar (23.4%), Ragnigadha (42.1%), Safal (46.7%), Meenajee (17.8%), Kamal Pasand (11.2%), Baba (9.3%), Signature (19.6%), and Tulsi (20.6%) were prominently displayed, indicating potential violations of regulations prohibiting tobacco advertising (Table 4).

**Table 4:** Presence of brand names in tobacco vendor (direct and indirect advertisement).

Variables	Name of the tobacco brand	Govt schools (N)	(%)	Private schools (n)	(%)	Total (n)	(%)
Direct advertisement	Gold flake	7	13	9	17	16	15
	Marlboro	11	20.4	14	26.4	25	23.4
	Classic	8	14.8	9	17	17	15.9
	Total	4	7.4	3	5.7	7	6.5
	Wills	2	3.7	2	3.8	4	3.7
	Four square	3	5.6	6	11.3	9	8.4
Indirect advertisement	Vimal	2	3.7	2	3.8	4	3.7
	Bahar	16	29.6	16	30.2	32	29.9
	Pan bahar	10	18.5	15	28.3	25	23.4
	Ragnigadha	22	40.7	23	43.4	45	42.1
	Safal	24	44.4	26	49.1	50	46.7
	Meenajee	9	16.7	10	18.9	19	17.8
	Kamal pasand	3	5.6	9	17	12	11.2
	Baba	5	9.3	5	9.4	10	9.3
	Signature	11	20.4	10	18.9	21	19.6
	Tulsi	9	16.7	13	24.5	22	20.6

### 3.6 Comparison between government and private schools

There was no significant difference between government and private schools with regard to various sections of the COTPA.

## 4 Discussion

### 4.1 Regulatory violations and tobacco vendor density

The findings highlight the alarming prevalence of tobacco vendors near schools, with widespread violations of tobacco control regulations. The high density of tobacco vendors within a 100-meter radius of schools raises serious concerns, as it increases the accessibility and visibility of tobacco products to students, a vulnerable population susceptible to tobacco use initiation (24). This proximity violates COTPA regulations, prohibiting the sale of tobacco products within a 100-yard radius of educational institutions (25). The study found an average tobacco vendor density of approximately seven within 100 yards of schools in Bhubaneswar City. Similar studies in Ranchi and Siliguri reported six and five vendors per square kilometer, respectively (26). This widespread presence of tobacco vendors near schools demonstrates the urgent need for stricter enforcement to protect students from early tobacco exposure.

### 4.2 International tobacco control measures and their relevance

Effective tobacco control measures, such as comprehensive smoke-free policies in public places, including educational institutions, have significantly reduced secondhand smoke exposure and tobacco use in countries like Australia, Canada, and the United Kingdom (27–29). Similarly, initiatives such as increased tobacco taxation, plain packaging regulations, and impactful anti-tobacco mass media campaigns have successfully reduced tobacco consumption, particularly among youth (30). While these measures have demonstrated success internationally, their implementation in India requires a context-specific approach considering socio-economic and cultural factors. Unlike high-income countries where strong enforcement mechanisms and widespread public health awareness campaigns support compliance, India faces challenges such as weaker regulatory oversight, economic reliance on the tobacco industry, and varying levels of law enforcement efficiency across states (31). Adapting global best practices, such as strict enforcement of tobacco-free zones, graphic health warnings, and large-scale awareness campaigns, could significantly strengthen India’s existing tobacco control framework.

### 4.3 Marketing strategies and tobacco advertising

The pervasive advertising and promotional activities near schools further exacerbate the problem. Vendors used various advertising methods, including boards, posters, banners, stickers, dangles, and product displays, many violating COTPA regulations (32, 33). The tobacco industry frequently employs aggressive marketing strategies such as the prominent display of brand names, distinctive color schemes, and specific layouts, which influence youth tobacco initiation. Studies have shown that increased exposure to tobacco advertising leads to higher initiation rates among adolescents, reinforcing the need for stricter enforcement of advertising bans (32, 33). Despite legal restrictions, the presence of such marketing techniques suggests a failure in enforcing tobacco control policies, necessitating stronger regulatory measures.

### 4.4 Health implications and youth exposure

The prevalence of smoking in public places near schools remains a significant public health concern. The study revealed that a majority of vendors, approximately 57.9%, allowed smoking within 100 yards of schools, exposing students and the general public to secondhand smoke, which is a well-established risk factor for respiratory diseases and cardiovascular conditions (34). Additionally, the absence of mandated health warning messages and signage near educational institutions represents a serious gap in compliance efforts (35, 36). The sale of tobacco products to minors is another critical concern, with nearly half of the vendors selling tobacco to minors, while a significant proportion had minors engaged in tobacco sales (37). These violations not only breach COTPA Section 6 but also contribute to early tobacco addiction and long-term health consequences (37). The presence of tobacco vendors near schools is also a violation of the Juvenile Justice Act, as it facilitates the sale of tobacco products to minors, a practice strictly prohibited under the law (38).

### 4.5 Policy interventions and enforcement strategies

Some states in India, such as Bihar, have demonstrated notable success in enforcing Tobacco-Free Educational Institution (TOFEI) guidelines, setting an example that Odisha could follow (39). Strategies involving regular compliance monitoring, strict penalties for violations, and collaboration with school authorities and community leaders have significantly improved implementation (40, 41). Training programs developed by the National Council of Educational Research and Training (NCERT) and directives from the Central Board of Secondary Education (CBSE) play a crucial role in educating teachers and school administrators about the importance of maintaining a tobacco-free environment (42, 43). Furthermore, intersectoral coordination between health, education, law enforcement, and civil society sectors, facilitated by bodies like the Tobacco Control Cell, is essential for ensuring the effective implementation of tobacco control laws (40).

To address these challenges, it is necessary to strengthen the enforcement of existing tobacco control laws, including COTPA and the Juvenile Justice Act, through regular monitoring and stringent penalties for violations (40). Implementing stricter regulations to completely eliminate tobacco advertising, promotion, and sponsorship, in line with the WHO Framework Convention on Tobacco Control (FCTC), can help reduce the influence of tobacco marketing on youth (44, 45). Additionally, enhancing community education and awareness campaigns, mainly targeting youth and their guardians, is crucial for preventing tobacco initiation and encouraging cessation (46).

### 4.6 Strengths of the study

This study has several key strengths that enhance its contribution to tobacco control research. It employs a rigorous methodology with a probability-proportional-to-size (PPS) sampling method, ensuring representative school selection in Bhubaneswar. The use of ArcGIS 10.8, Google Maps, and satellite imagery enhances the accuracy of vendor mapping within a 100-yard radius, providing quantitative evidence on vendor clustering and its potential impact on youth tobacco exposure. As one of the first studies to explore tobacco vendor density near schools in Odisha, it provides region-specific insights to inform state-level policy interventions. Additionally, it evaluates compliance with COTPA regulations (sections 4, 5, and 6), shedding light on gaps in enforcement, tobacco advertising violations, and sales to minors. Furthermore, the multi-dimensional analysis of tobacco marketing strategies, including direct and indirect advertising, highlights the tobacco industry’s influence on youth tobacco initiation. These findings provide data-driven insights for strengthening tobacco-free school policies, zoning laws, and vendor regulations, positioning this research as a valuable resource for policymakers, public health officials, and researchers working to enhance tobacco control efforts in India.

### 4.7 Limitations

The study has several limitations that warrant consideration. Firstly, the sample size was limited to 15 high schools in Bhubaneswar City, Odisha, which could constrain the applicability of the findings to other regions. Additionally, the cross-sectional design used in the study provides a static view and may not capture dynamic changes or trends over time. Reliance on observational assessments introduces the possibility of reporting bias, potentially impacting the accuracy of compliance levels or vendor practices reported. Furthermore, the study’s focus on specific variables related to tobacco control near schools may overlook broader contextual factors and socioeconomic influences that could significantly influence tobacco use initiation among youth. These limitations highlight the need for future research with larger and more diverse samples, longitudinal designs, and comprehensive assessments of contextual factors to achieve in-depth understanding of tobacco control dynamics.

## 5 Conclusion

This study reveals a concerning landscape of widespread tobacco vendor density and regulatory non-compliance in the vicinity of educational institutions. The presence of numerous tobacco vendors within a 100-meter radius of schools, coupled with the pervasive display of tobacco advertisements and the sale of tobacco products to minors, highlights significant gaps in the implementation and enforcement of tobacco control regulations. These findings emphasize the urgent need for stronger policy interventions and consistent enforcement mechanisms to curb youth access to tobacco products. Additionally, community-driven initiatives and grassroots advocacy can play a pivotal role in strengthening local tobacco control efforts.

Efforts should be directed toward enhancing awareness campaigns, mainly targeting youth and their guardians. Involving various stakeholders, such as educational institutions, community leaders, and civil society organizations, and fostering intersectoral collaborations in tobacco control initiatives can foster a supportive environment for tobacco cessation and prevention. All stakeholder departments and enforcers should make concerted effort to protect the youth from exposure and use of tobacco products.

By addressing the multifaceted issues highlighted in this study, progress can be made in reducing the burden of tobacco-related morbidity and mortality, particularly among vulnerable populations like youth. Implementing a combination of strict regulatory enforcement, public health education, and continuous surveillance can create long-term, sustainable reductions in youth tobacco exposure and consumption.

## Data Availability

The original contributions presented in the study are included in the article/Supplementary material, further inquiries can be directed to the corresponding author.
