# Supplementary material for: Vendor density mapping and compliance assessment with tobacco control laws around schools in Bhubaneswar City—a geo-spatial mapping and observational study
Source: Front Public Health. 2025 Jun 13;13:1410114. doi: 10.3389/fpubh.2025.1410114 (PMC12202329; doi:10.3389/fpubh.2025.1410114)
Supplement: Supplementary file 1 [file Table_1.docx]

Supplementary File 1: Overview of Variables, Measures, and References for Compliance Assessment in Tobacco Vendor Environments

| Variable | Measure | Reference |
| --- | --- | --- |
| Vendor Type | Type of vendor (e.g., Pan vendors, Tea stall, Grocery/convenience store, large store/supermarket, Mobile vendor) | GATS and GYTS |
| Presence of Smoking in Public Places | Presence of smoking in public places | COTPA Section 4 |
| Advertisements in Tobacco vendors/shops | Presence advertisements in tobacco vendors/shops | COTPA Section 5 |
| Type of Advertisements - Boards | Type of material: Signs | COTPA Section 5 |
|  |  |  |
| Type of Advertisements - Posters | Type of material: Displays | COTPA Section 5 |
| Type of Advertisements - Banners | Type of material: Displays | COTPA Section 5 |
| Type of Advertisements - Stickers | Type of material: Displays | COTPA Section 5 |
| Type of Advertisements - Dangles | Type of material: Displays | COTPA Section 5 |
| Type of Advertisements - LCD/Video Screening/LED | Type of material: Displays | COTPA Section 5 |
| Type of Advertisements - Promotional gifts/offers | Type of material: Displays | COTPA Section 5 |
| Type of Advertisements - Product display | Type of material: Displays | COTPA Section 5 |
| Advertisement board displays brand pack shot or brand name of tobacco products | Requirement for health warning on advertisement boards | COTPA Section 5 |
| Presence of Hoarding advertising tobacco products | Presence of hoarding advertising tobacco products | COTPA Section 5 |
| Brand Names Displayed | Presence of specific tobacco brand names displayed | COTPA Section 5 |
| Indirect Advertisements in Tobacco Shops/vendors | Presence indirect advertisements in tobacco shops/vendors | COTPA Section 5 |
| Brand Names Displayed (Indirect) | Presence of specific tobacco brand names displayed | COTPA Section 5 |
| Health Warning Messages | Presence of board/banner/poster displays with health warning | COTPA Section 5 |
| Whether health warning is on uppermost portion of a board | Requirement for health warning placement on advertisement boards | COTPA Section 5 |
| Whether health warning is written in any local Indian language (and/or English) | Requirement for health warning language on advertisement boards | COTPA Section 5 |
| Advertisement Location: Exteriors | Number of materials located outside the store, including windows, doors, building, sidewalk, or parking lot | Feighery et al. (2001) |
| Advertisement Location: Interior | Number of materials located inside the store, including those attached to the window or door | Feighery et al. (2001) |
| Advertisements Placement below 3 feet | Presence of advertising materials at or below 3 feet from the floor | Feighery et al. (2001) |
| Advertisements Placement next to candy | Presence of displays within 6 inches of candy (sweets) | Feighery et al. (2001) |
| Advertisements Placement above 3 feet | Presence of advertising materials above 3 feet from the floor | Feighery et al. (2001) |
| Display of signage as mandated in law | Presence of signage stating prohibition of tobacco sales to minors | COTPA Section 6(a) |
| Tobacco products are sold by minors | Presence of tobacco products sold by minors | COTPA Section 6(a) |
| Tobacco products are sold to minors | Presence of tobacco products sold to minors | COTPA Section 6(a) |
| Tobacco Warning Signage in Public Places | Presence of Signage around 100 yards of educational institution | COTPA Section 6 |
