# Supplementary figures and images for: Vendor density mapping and compliance assessment with tobacco control laws around schools in Bhubaneswar City—a geo-spatial mapping and observational study
Source: Front Public Health. 2025 Jun 13;13:1410114. doi: 10.3389/fpubh.2025.1410114 (PMC12202329; doi:10.3389/fpubh.2025.1410114)

Supplementary file 2: Depiction of Various Instances of Non-Compliance


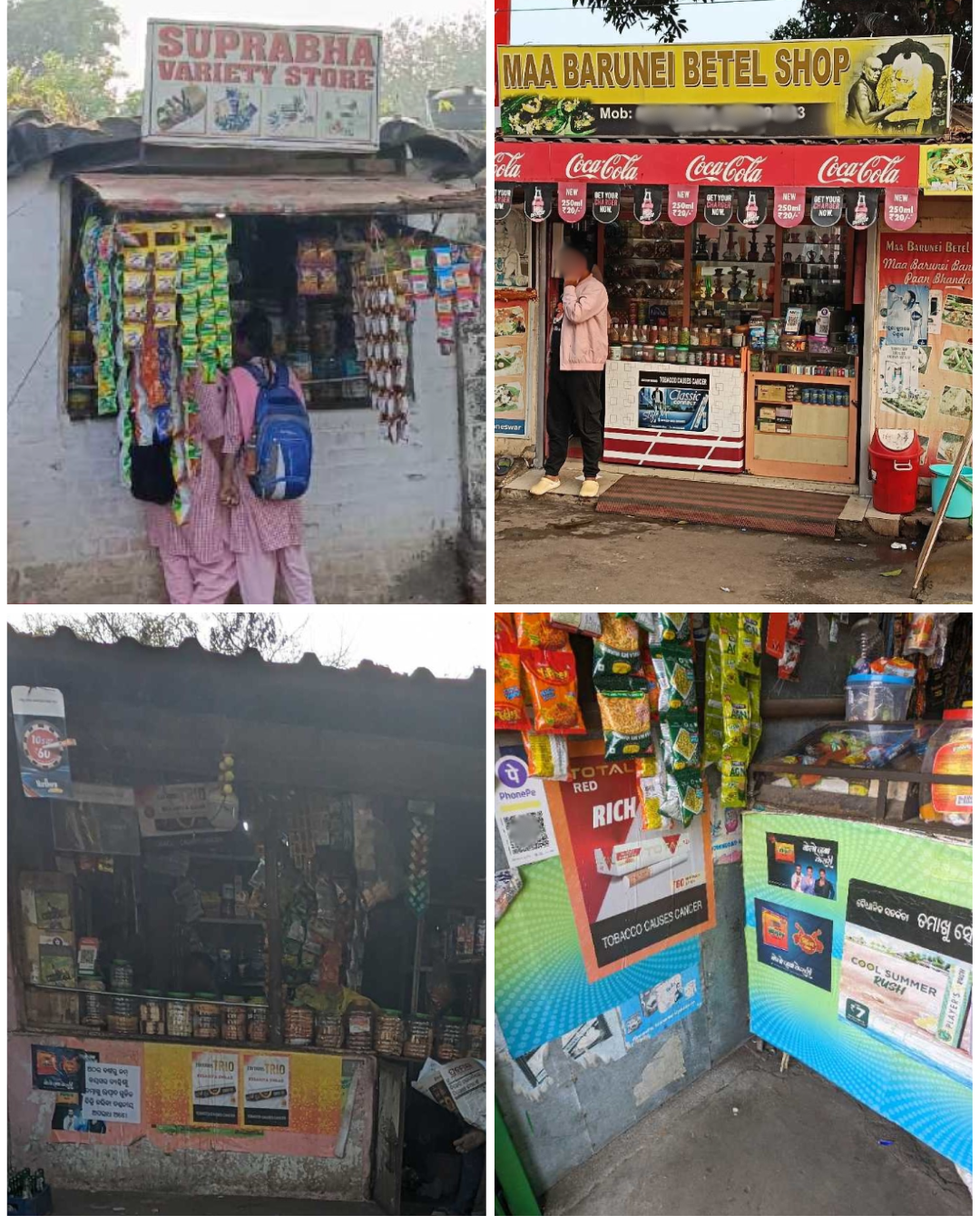

Supplement: Supplementary file 2 [file Table_2.docx]
